# Diffusion MRI in Evaluation of Pediatric Posterior Fossa Tumors

**DOI:** 10.31557/APJCP.2021.22.4.1129

**Published:** 2021-04

**Authors:** Warinthorn Phuttharak, Mix Wannasarnmetha, Sakda Waraaswapati, Sopiruch Yuthawong

**Affiliations:** 1 *Department of Radiology, Faculty of Medicine, Khon Kaen University, Khon Kaen, Thailand. *; 2 *Department of Pathology, Faculty of Medicine, Khon Kaen University, Khon Kaen, Thailand. *

**Keywords:** Pediatrics, posterior fossa, brain neoplasms, medulloblastoma, diffusion MRI

## Abstract

**Background:**

To evaluate the role of diffusion MRI in differentiating pediatric posterior fossa tumors and determine the cut-off values of ADC ratio to distinguish medulloblastoma from other common tumors.

**Methods:**

We retrospectively reviewed MRI of 90 patients (7.5-year median age) with pathologically proven posterior fossa tumors (24 medulloblastoma, 7 ependymoma, 4 anaplastic ependymoma, 13 pilocytic astrocytoma, 30 diffuse intrinsic pontine glioma (DIPG), 4 ATRT, 3 diffuse astrocytoma, 2 high grade astrocytoma, 2 glioblastoma, and 1 low grade glioma). The conventional MRI characteristics were evaluated. Two readers reviewed DWI visual scale and measured ADC values by consensus. ADC measurement was performed at the solid component of tumors. ADC ratio between the tumors to cerebellar white matter were calculated.

**Results:**

The ADC ratio of medulloblastoma was significantly lower than ependymoma, pilocytic astrocytoma and DIPG. The ADC cut-off ratio of ≤ 1.115 allowed discrimination medulloblastoma from other posterior fossa tumors with sensitivity, specificity, PPV and NPV of 95.8%, 81%, 67.6% and 97.9%, respectively. ADC ratio cut-off level to differentiate medulloblastoma from ependymoma was ≤ 0.995 with area under the curve (AUC)= 0.8693. ADC ratio cut-off level for differentiate medulloblastoma from pilocytic astrocytoma at ≤ 1.17 with AUC = 0.9936. ADC cut-off level for differentiate medulloblastoma from DIPG at ≤ 1.195 with AUC = 0.9681. The ADC ratio was correlated with WHO grading by the lower ADC ratio associated with the higher grade. Furthermore, High DWI visual scale was associated with high grade tumor.

**Conclusion:**

Diffusion MRI has a significant role in diagnosis of pediatric posterior fossa tumors. ADC ratio can be used to distinguish medulloblastoma from other posterior fossa tumor with good level of diagnostic performance.

## Introduction

Central nervous system tumor account for 20% of all tumors among pediatric patients and posterior fossa is the most frequent location in populations older than 1 year of age, but cerebral hemispheres is the common location for adult patients.(Helton et al., 2008) Variety of tumor types arising in posterior fossa are classified by WHO grading according to histologic subtype and degree of anaplasia (Helton et al., 2008; Louis et al., 2007) and also these gradings indicate the prognostic factor of the patients (Louis et al., 2007)

The common pediatric posterior fossa tumors are comprised of pilocytic astrocytoma, medulloblastoma, ependymoma, diffuse intrinsic pontine glioma (DIPG) and a rare disease, atypical teratoidrhabdoid tumor (ATRT). The highest WHO grading and the most common malignant tumor is medulloblastoma as Aquilina (2013) reported which the treatment should be more aggressive for the best outcome as Bergman (2019) reported in their previous works. Medulloblastoma, as the highest grade of malignancy, has histological feature as high cellularity of the tumor (Louis et al., 2007). Although these tumors can usually be differentiated by their characteristic features in conventional MRI findings as Aquilina (2013) reported, the diagnosis sometimes cannot be made correctly by lack of typical features in the tumors. Heterogeneity in imaging appearance limits confident diagnostic decision in many cases, especially the three most common pediatric posterior fossa tumors; medulloblastoma, ependymoma, and cerebellar astrocytoma. Distinction of medulloblastoma from ependymoma could be challenging because of their typical fourth ventricular location.

Even histopathology remains the gold standard for definite diagnosis, but there are some limitations of sampling errors during biopsy from tumor heterogeneity where tumor under grading can occur. It has been reported (Bihan, 2003) as diffusion MRI is the non-invasive tool to measure water diffusion within the lesion which could demonstrate whether the lesion has high or low cellularity. Diffusion weighted imaging (DWI) has been explored as a rapid method for grading of brain tumors, either by visual assessment of signal characteristics or quantitative analysis of apparent diffusion coefficient (ADC) values (Seo et al., 2008; Phuttharak et al., 2020). Unfortunately, some of the previous studies showed overlapping of ADC value between different grades and types of tumor, which is too great to specifically diagnose individual brain tumors with DWI alone (Kan et al., 2006; Schneider et al., 2007). In contrast, there were more studies which suggested DWI may be highly accurate in tumor diagnosis in pediatric posterior fossa (Rumboldt et al., 2006; Jaremko et al., 2010; JI et al., 2011; Ahmed et al.,2018; Zitouni et al., 2017; Pierce et al., 2014; Lemeshow et al, 1990). One study showed no overlapping between ADC values in the three main pediatric posterior fossa tumors: medulloblastoma, pilocytic astrocytoma (JPA) and ependymoma (Rumboldt et al., 2006). However, there are still some discrepancies.

Thus, the purpose of this study was to determine whether diffusion MRI could help to differentiate the posterior fossa tumor in children by using ADC ratio and whether there is a correlation between visual scale on DWI and WHO tumor grading.

## Materials and Methods

*Study population*

This study was a retrospective study and conducted at Department of Radiology, Srinagarind Hospital, Faculty of Medicine, Khon Kaen University, Thailand. The inclusion criteria were pediatric patients less than 20 years of age with posterior fossa tumor who had performed MRI and DWI/ADC prior to any treatment. The study period was between January 2008 and June 2019. The study protocol was approved by the ethic committee in human research, Khon Kaen University. Informed consents were not required by the approval of the ethic committee.

*Imaging technique*

Using a routine brain protocol (Sagittal 3D TFE T1W imaging, Ax TSE T2W and 3D FLAIR imaging, Coronal T2W gradient/Ax SWI and post contrast axial, coronal and sagittal imaging); 3D TFE T1W images (8.6/4.1; number of signal acquired, 1 mm; section thickness, 5 mm; intersection gap, 1 mm; matrix, 256x256; field of view [FOV], 23 x 23 cm), TSE T2W images (4,500/96; number of signal acquired,1; section thickness, 5 mm; intersection gap, 1 mm; matrix, 400x270; FOV, 23 x 20 cm). T1-weighted fat-suppressed gradient echo sequences after administration of gadolinium contrast 0.1 mmol/kg (Gadobutrol, Gadovist; Bayer Healthcare Pharmaceuticals) was also performed as part of the routine protocol.

All patients underwent DWI/ADC with either a 1.5T (Siemens Magnetom aera; Siemens Healthcare, Erlangen, Germany) or 3T MR (Phillips Achieva dStream; Philips, Best, the Netherlands) scanners. A single shot echo-planar diffusion-weighted imaging sequence was performed. Imaging parameters of DWI were as followings: 3,000-45,00/89-95 (TR/TE) with diffusion sensitivities b=0 and b=1,000 s/mm2 for both scanners. The diffusion gradients were applied sequentially in three orthogonal directions to generate 2 sets of axial DW images. The ADC maps were automatically generated from the datasets of DWI images using the operating console and ADC were calculated.

*Post-Processing*

DWI data were transferred to a Synapse 3D workstation (Fujifilm Medical Systems, USA, Inc.) and ADC maps were generated. 

*Imaging analysis*

MRI were reviewed by two experienced neuroradiologists who were blinded to the patient’s pathological diagnosis on Picture Archiving and Communication System (PACS) by consensus manner, as the following details.

*i. Conventional MRI characteristics*

All of the imaging were analyzed in conventional MRI characteristics on T1W, T2W, FLAIR, Gradient/SWI and post contrast study. The score of signal intensity on T1W and T2W images were classified into 5 scales (for T1W score; score 1: Higher than white matter, score 2: Isointense to white matter, score 3: As low as gray matter, score 4: Higher than CSF, lower than gray matter, score 5: As low as CSF, and for T2W score; score 1: Lower than white matter, score 2: As low as white matter, score 3: Isointense to gray matter, score 4: Lower than CSF, higher than gray matter, score 5: As high as CSF). 

The location of the tumors was classified as midline and lateral location. Cystic component was the area within the tumor that show water signal intensity with smooth border and no rim enhancement and was classified into macrocystic (more than or equal to 1 cm in size) and microcystic (less than 1 cm in size). Necrotic area was the non-cystic and non-enhancing tissue within the tumor which was distinguished from cystic area by its irregular border and lack of rim enhancement and then was classified into presence and absence. Enhancement was graded by the percent of area enhancement (Moderated to marked: more than 25 percent of the tumor, minimal: less than 25 percent of the tumor, absent: no enhancement, peripheral: rim enhancement only).

Grading area of high signal intensity on T2w/FLAIR images of the peritumoral structure (None, mild, extensive) was also performed by using visual scale.

Calcification or blood components containing within the tumor was defined by the present area of very low signal intensity on T1W, T2W, FLAIR images and susceptibility artifact on gradient images/SWI (present, absent).

*ii. Diffusion imaging*

The ADC value obtained at the solid part with greatest restricted diffusion on ADC mapping by avoiding the area of necrosis, bleeding and calcification. The regions of interests were placed in three different locations, the mean ADC value was calculated. Ratio between the mean lowest ADC value within the solid tumor to normal cerebellar white matter was calculated.

Visual scale on DWI images was grading by signal intensity on DWI images of the highest b-value at the highest restricted diffusion area within tumor by comparison to adjacent normal gray matter of cerebellum using five-point scale (-2: markedly hypointense, -1: hypointense, 0: isointense, 1: hyperintense, 2: markedly hyperintense).

*Statistical analysis*

All data were analyzed using SPSS statistics Version 19.0.2. Descriptive statistics was used to describe the demographic data and conventional MRI findings, also with T1W and T2W signal intensity score. Categorical data was demonstrated as number and percentage, continuous data was demonstrated as mean, standard deviation, range, and median.

Comparison of the ADC ratio in different types of tumor was compared by Kruskal-Wallis test with Bonferroni corrected significant level at p-value < 0.00139. Comparison of the ADC ratio and DWI visual scale between high- and low-grade tumors were compared by Mann-Whitney U test with significant level at p-value < 0.05.

The optimal cut-off level of ADC ratio and DWI visual scale to differentiate medulloblastoma from other posterior fossa tumors and to differentiate high grade tumor from low grade tumor were analyzed by using logistic regression analysis and ROC curves.

## Results

*Demographic Data*

During the study period, there were 100 patients with posterior fossa tumors, but 10 patients were excluded as lack of adequate tumor size for measurement. Finally, the population in this study was 90 cases. Of those, the histopathological findings were 24 medulloblastoma, 7 WHO II ependymoma, 4 WHO III anaplastic ependymoma, 13 pilocytic astrocytoma, 30 diffuse intrinsic pontine glioma (DIPG), 4 ATRT, 3 WHO II diffuse astrocytoma, 1 WHO III anaplastic astrocytoma, 1WHO IV astrocytoma, 2 WHO IV glioblastoma and 1 low grade glioma(Table1). All cases had pathological diagnosis except diffuse intrinsic pontine glioma (DIPG) which was diagnosed on the basis of conventional MRI findings. The patients were divided into two main groups as high grade (WHO III-IV) and low grade (WHO I-II) groups. The median age of all patients was 7.5 years (range 1-19) with somewhat higher proportion of male sex (52.22%) (Table1).

*Conventional MRI characteristics*

Most cases of medulloblastoma showed isointensity on T1WI (62.5%), hyperintensity on T2WI and FLAIR (70.83%), midline location (83.33%), more than 25% enhancement (91.67%), mild peritumoral edema (79.17%), presence of necrotic area (62.5%) and calcification/blood component (79.17%). T1W signal intensity score 3 (isosignal intensity to gray matter) contained 15 medulloblastoma (62.5%), 9 ependymoma (81.82%), 2 DIPG (6.62%) and 1 ATRT (25%). For T2W signal intensity score, most tumors showed score 4 (higher signal intensity than gray matter but less than CSF) with all 11 cases of ependymoma showed score 4 on T2W images. Seven cases of pilocytic astrocytoma (53.85%) show score 5 (signal intensity equal to CSF) on T2W images.

*Diffusion MRI*

**Figure 1:**
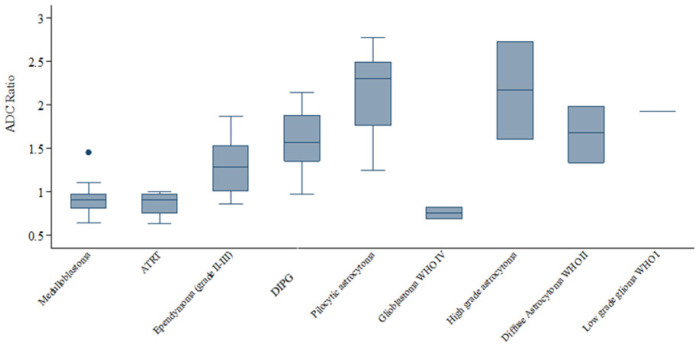
Distribution of ADC Ratio in Different Tumor Types: The limit of the box represents the lower quartile (25^th ^percentile) and upper quartile (75^th^ percentile); the length of the box height is the interquartile range. The top and bottom whiskers are the maximum and minimum value. The line dividing the box is the median

For diffusion imaging, ADC ratio of tumors were shown in table 2, The distribution of ADC ratio in different tumor types was shown in Figure 1. 

The ADC ratio of medulloblastoma was significantly lower than DIPG, ependymoma and pilocytic astrocytoma with p-value of <0.00139, 0.0005, and <0.00139, respectively but was not significantly different from ATRT, glioblastoma, high grade astrocytoma, diffuse astrocytoma, and low grade glioma with p-value of 0.8438, 0.1356, 0.0209, 0.0069 and 0.0959, respectively. The ADC ratio of ependymoma was significantly different from pilocytic astrocytoma (p-value = 0.0013). 

The ADC ratio between high grade and low-grade groups were significantly different with 1.28 ± 0.46 versus 1.78 ± 0.57, respectively (p-value< 0.001). 

**Figure 2:**
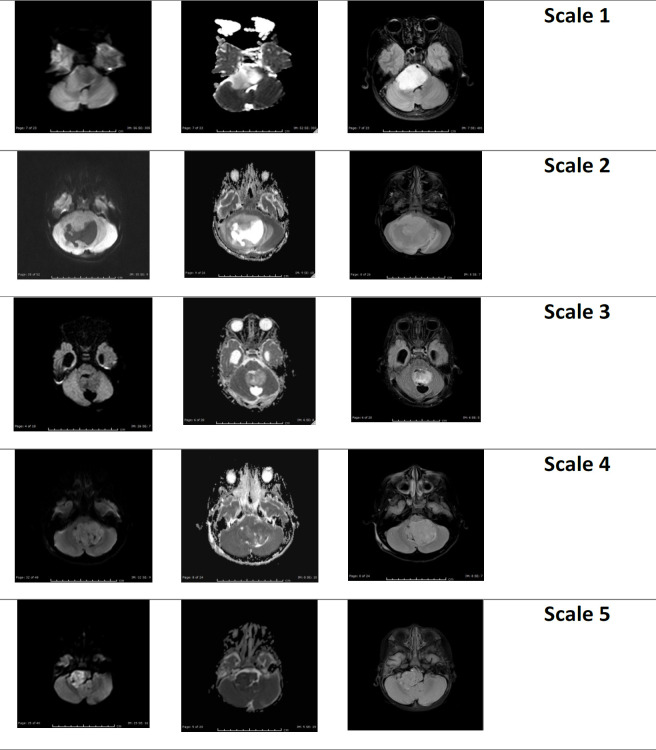
Different DWI Visual Scale. A, DWI visual scale 1; B, DWI visual scale 2; C, DWI visual scale 3; D, DWI visual scale 4; E, DWI visual scale 5

 The DWI visual scale of the tumors showed that the score 5 contained 18 medulloblastoma (75%), 5 ependymoma (45.45%), 1 DIPG (3.33%) and 4 ATRT (100%), as shown in Table 2. The types of DWI visual scale were shown in Figure 2. The DWI visual scale of low grade and high-grade tumors showed significant difference at score 5 (p-value = 0.016) as shown in Table 2. 

*ADC ratio and DWI visual scale cut-off level*

**Figure 3:**
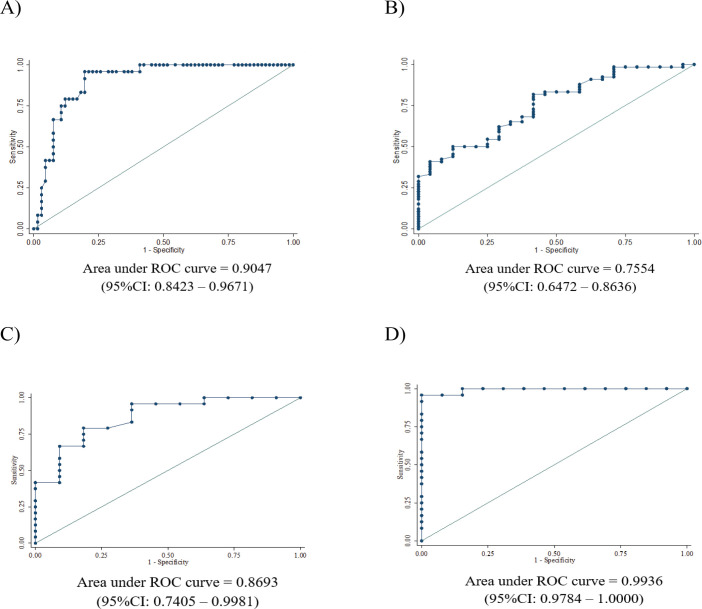
ROC Curves of ADC Ratio Cut Off Levels for Different Tumor Type Differentiation. A, Medulloblastoma from other high grade tumors; B, High grade from low grade tumors; C, Medulloblastoma from ependymoma; D, Medulloblastoma from pilocytic astrocytoma; E, Medulloblastoma from DIPG; F, Medulloblastoma from ATRT

The ROC analysis showed that ADC ratio cut-off level of ≤ 1.115 can differentiate medulloblastoma from other posterior fossa tumor with sensitivity, specificity, PPV and NPV being 95.8%, 81%, 67.6% and 97.9%, respectively. The ADC ratio cut-off level for differentiation medulloblastoma from ependymoma, medulloblastoma from ATRT, medulloblastoma from pilocytic astrocytoma and medulloblastoma from diffuse midline glioma were demonstrated in Table 3, the ROC curves of these cut-off levels were shown in Figure 3. The ADC ratio cut-off level of ≤ 1.67 can diagnose high grade tumor with sensitivity, specificity, PPV and NPV being 81.8%, 58.3%, 84.4% and 53.8%, respectively and area under the curve (AUC) was 0.7554. ADC ratio cut-off level to differentiate medulloblastoma from other tumors was ≤ 1.115 and AUC was 0.9047. ADC ratio cut-off level to differentiate medulloblastoma from ependymoma was ≤ 0.995 and AUC was 0.8693. ADC cut-off level for differentiate medulloblastoma from pilocytic astrocytoma at ≤ 1.17 with AUC being 0.9936. ADC cut-off level for differentiate medulloblastoma from DIPG at ≤ 1.195 with AUC being 0.9681.

The DWI visual scale cut-off level of ≥ 4.5 for differentiation medulloblastoma from other posterior fossa tumor generated sensitivity, specificity, PPV and NPV as 75%, 83.3%, 62.1% and 90.2%, respectively). The cut point of DWI visual scale of ≥ 3.5 for diagnosis high grade tumor with sensitivity, specificity, PPV and NPV being 69.7%, 66.7%, 85.2% and 44.4%, respectively).

**Table 1:** Demographic Data

	Overall (n=90)	High Grade (n=66)		Low Grade (n=24)
		Medulloblastoma	Other high-grade tumor	
		(n=24)	(n=42)	
Age (years)				
Median (min-max)	7.5 (1 - 19)	8 (1 - 19)	6.5 (1 - 17)	9 (1 - 18)
Median (IQR)	7.5 (3 - 11)	8 (5 - 12)	6.5 (4 - 11)	9 (3 - 11)
Mean	7.94 (4.99)	8.63 (5.11)	7.69 (4.9)	7.71 (5.17)
Sex				
Male	47 (52.22)	17 (70.83)	17 (40.48)	13 (54.17)
Female	43 (47.78)	7 (29.17)	25 (59.52)	11 (45.83)

**Table 2:** Types of Posterior Fossa Tumor

Tumor	Number
High grade tumor	
Medulloblastoma	24
Diffuse midline glioma	30
Anaplastic ependymoma	4
ATRT	4
Anaplastic astrocytoma	1
High grade, unclassified astrocytoma	1
Glioblastoma	2
Low grade tumor	
Ependymoma	7
Pilocytic astrocytoma	13
Diffuse Astrocytoma	3
Low grade glioma	1

**Table 3:** ADC Ratio and DWI Visual Scale of Tumors

	ADC Ratio			DWI visual Scale [number of tumor(percentage)]				
	Mean ± SD	Min	Max	1	2	3	4	5
1- Medulloblastoma (n=24)	0.91 ± 0.17	0.64	1.45	0 (0)	0 (0)	0 (0)	6 (25)	18 (75)
2- ATRT (n=4)	0.86 ± 0.16	0.63	1	0 (0)	0 (0)	0 (0)	0 (0)	4 (100)
3- Ependymoma (grade II-III) (n=11)	1.3 ± 0.35	0.86	1.87	0 (0)	0 (0)	2 (18.18)	4 (36.36)	5 (45.45)
4- DIPG (n=30)	1.57 ± 0.32	0.97	2.14	1 (3.33)	6 (20)	12 (40)	10 (33.33)	1 (3.33)
5- Pilocytic astrocytoma (n=13)	2.11 ± 0.51	1.24	2.77	0 (0)	7 (53.85)	5 (38.46)	1 (7.69)	0 (0)
6- Glioblastoma WHO IV	0.75 ± 0.1	0.68	0.82	0 (0)	0 (0)	1 (50)	0 (0)	1 (50)
7- High grade astrocytoma (n=2)	2.17 ± 0.8	1.6	2.73	0 (0)	1 (50)	0 (0)	1 (50)	0 (0)
8- Diffuse Astrocytoma WHO II (n=3)	1.66 ± 0.33	1.33	1.98	0 (0)	0 (0)	1 (33.33)	2 (66.67)	0 (0)
9- Low grade glioma WHO I (n=1)	1.92	1.92	1.92	0 (0)	0 (0)	1 (100)	0 (0)	0 (0)

**Figure 4:**
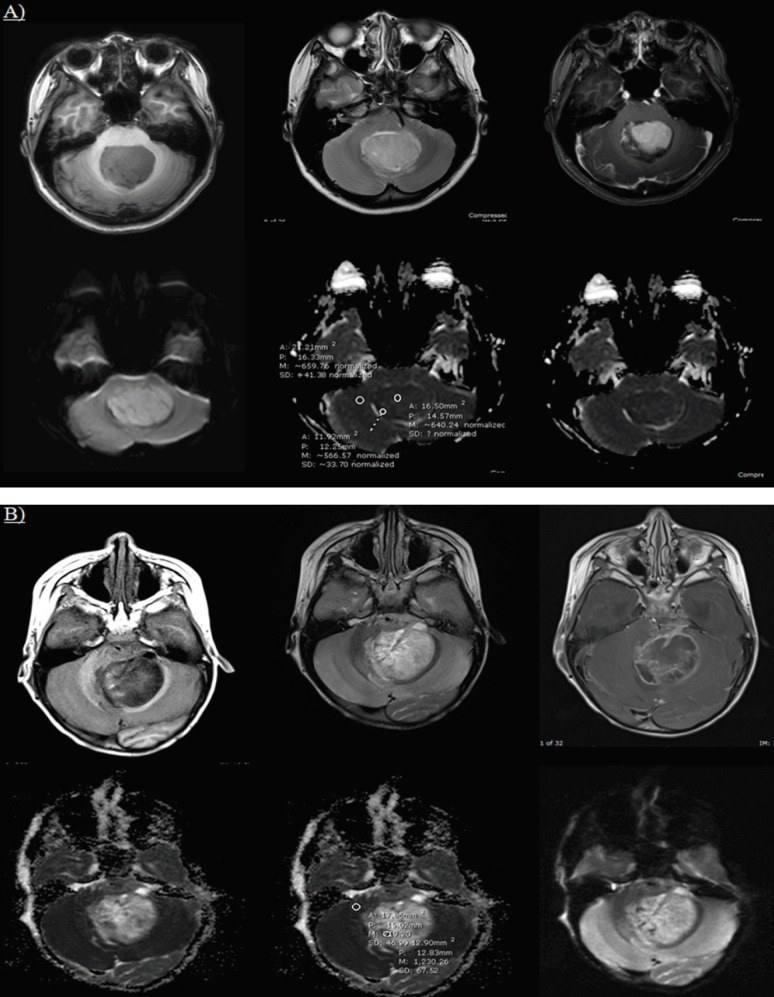
ADC Map Images of Medulloblastoma and Ependymoma Cases who had Overlapped Conventional MRI Findings by Using the ADC Ratio Cut-Off Level <1.18 for Tumor Differentiation. A, Medulloblastoma with T1W signal intensity score 3 (isointensity), ADC ratio was 0.92 and score 5 of DWI visual scale; B, Ependymoma with with T1W signal intensity score 3 (isointensity), ADC ratio was 1.87 and score 5 of DWI visual scale

**Table 4:** ADC Ratio Cut-Off Level

	ADC ratio	Sensitivity	Specificity	PPV	NPV
Med Vs others	≤ 1.115	95.8	81	67.6	97.9
Med Vs Epen	≤ 0.995	79.2	81.8	90.5	64.3
Med Vs ATRT	≤ 0.935	66.7	50	88.9	20
Med Vs PA	≤ 1.17	95.8	100	100	92.9
Med Vs DIPG	≤ 1.195	95.8	90	88.5	96.4

** Med, medulloblastoma; Epen, Ependymoma; ATRT, atypical teratoid rhabdoid tumor; PA, pilocytic astrocytoma; DIPG, diffuse intrinsic pontine glioma; PPV, positive predictive value; NPV, negative predictive value

## Discussion

Pediatric brain tumors encompass a heterogeneous group of cell types. Treatment and prognosis vary with tumor cell types, tumor grading, staging, and extension of resection.

Diffusion MRI is a non-invasive tool to measure water molecule diffusivity which related to degree of the tumor’s cellularity. Both qualitative DWI assessment and quantitative ADC measurement could be easily applied. Recent studies showed that there was a correlation between the WHO grading of tumors and ADC value and quantitative assessment is more promising (Rumboldt et al., 2006; Jaremko et al., 2010; JI et al., 2011; Ahmed et al., 2018; Zitouni et al., 2017; Pierce et al., 2014; Lemeshow et al.,1990). Furthermore, ADC ratio of the tumor to normal cerebellar white matter for internal standardization could be simply done and able to be applied with MRI imaging from different MRI scanners.

In this study, we retrospectively evaluated the role of diffusion MRI in characterizing and differentiation pediatric posterior fossa tumors by correlated to pathological findings. Since MRI images in our study were performed from different MRI scanners, we used only ADC ratio in the quantitative measurement.

In the present study, results showed a significant difference between ADC ratio in different tumor grades, histological subgroups including the common three pediatric posterior fossa tumors: pilocytic astrocytoma, ependymoma, medulloblastoma. ADC ratio between high grade and low-grade groups were significantly different. This corresponded with previous study (Ahmed et al., 2017). This might be explained by the information that restricted diffusion from limited water molecular diffusivity representing high cellularity of the tumor. As highest WHO grade of ATRT, medulloblastoma and glioblastoma (WHO IV), ADC ratio showed the lowest value. Ependymoma as WHO grade II and III tumor had higher ADC ratio than medulloblastoma. Pilocytic astrocytoma as WHO grade I tumor had higher ADC ratio than ependymoma and medulloblastoma. Low- and high-grade tumor could be differentiated by using ADC ratio cut-off level ≤1.67 with fair level of diagnostic efficacy as sensitivity, specificity, PPV and NPV being 81.8%, 58.3%, 84.4% and 53.8%, respectively. Thus, the low sensitivity and specificity could be due to variety in histopathological appearances in various types of the tumor in each WHO groups, resulting in different ADC ratio of the tumors even in the same WHO grading. 

ADC ratios of medulloblastoma, ependymoma, pilocytic astrocytoma, DIPG and ATRT in our study were 0.91±0.17, 1.3±0.35, 2.11±0.51, 1.57±0.32 and 0.86±0.16, respectively which correlated to WHO grading of the tumors as higher grade showed lower ADC ratio and lower grade showed higher ADC ratio. Also, ADC ratio of medulloblastoma was significantly lower than ependymoma and pilocytic astrocytoma. ADC ratio of medulloblastoma was significantly lower than DIPG. These findings were consistent with several previous studies (Rumboldt et al., 2006; Jaremko et al., 2010; JI et al., 2011; Ahmed et al., 2018; Zitouni et al., 2017; Pierce et al., 2014; Lemeshow et al., 1990). ADC ratio was unable to differentiate medulloblastoma from ATRT, which is consistent with the study of Ahmed et al., (2018). The explanation of this could be from the similarity of medulloblastoma and ATRT in high grade according to WHO grading system representing high tumoral cellularity, thus, resulting in low ADC ratio. Cut-off level of ADC ratio to differentiate medulloblastoma from other posterior fossa tumors in our study was ≤1.115 with good level of diagnostic efficacy as sensitivity, specificity, PPV and NPV being 95.8%, 81%, 67.6% and 97.9%, respectively. ADC ratio cut-off level to differentiate medulloblastoma from ependymoma was ≤ 0.995 with good level of diagnostic performance as sensitivity, specificity being 79.2%, 81.8%, respectively, compared to findings from Soubhi Zitouni et al., (2017) which their study comprised of 14 cases of pilocytic astrocytoma, 10 cases of ependymoma and 18 cases of medulloblastoma, the ADC ratio cut-off level to differentiate medulloblastoma from ependymoma was ≤1.18 with sensitivity and specificity being 100% and 88.89%, respectively. The result from our study showed that ADC ratio range of ependymoma was lower than as reported by Soubhi Zitouni et al., (2017) which could be from the different number of cases of different grades of ependymoma as in our study (4 anaplastic ependymoma WHO grade III and 7 ependymoma WHO grade II, but they did not mentioned. ADC cut-off level at ≤1.17 could be differentiate medulloblastoma from pilocytic astrocytoma with excellent level of diagnostic performance from area under the curve (AUC) being 0.9936 in ROC curve. This corresponded with the study by Ahmed et al., (2018). ADC cut-off level at ≤ 1.195 also differentiated medulloblastoma from DIPG with very good level of diagnostic performance from AUC being 0.9681 in ROC curve. 

The conventional MRI findings of different types of tumor showed no significant different except the T1W score of ependymoma and medulloblastoma that tended to be score 3 (isosignal intensity to grey matter) with 9 cases of ependymoma (81.82%) and 15 of medulloblastoma (62.5%), these findings showed that there was some overlapping at this score. But the ADC ratio cut-off level at ≤1.18 could be applied to overcome this problem and able to differentiate these two tumor types.

The DWI visual scale showed no significant difference among tumor types but at the higher DWI visual scale, the higher grade the tumor tended to be corresponding with ADC ratio. At the score 5, there was significant difference between high grade and low-grade tumors. Then score 5 of DWI visual scale and low ADC ratio should raise the possibility of the tumor to be high grade.

Measurement of ADC is no longer time-consuming process. We can measure ADC quickly with routine image viewing software and no post processing is required. ADC ratio is a helpful measurement when dealing with MRI images from different MRI scanners. 

There were some limitations in our study. First, our study was a retrospective cross-sectional study. Second, the MRI images were from different MRI scanners which may influence ADC value measurement. However, we measured the ADC ratio of the tumor to normal cerebellar white matter in the same imaging of the patient which could be internal standardization. Third, a relatively small number of patients, particularly the limited number of cases in some histopathology subgroup.

In Conclusion, diffusion MRI has a significant role in diagnosis of various types of pediatric posterior fossa tumors. ADC ratio can be used to differentiate medulloblastoma from other posterior fossa tumor in pediatric patients with good level of diagnostic performance.

## Author Contribution Statement

All authors contributed to the study design. Material preparation, data collection, manuscript drafting were performed by Sopiruch Yuthawong, Warinthorn Phuttharak and Mix Wannasarnmetha did imaging data interpretation. Data analysis was collected and did the conclusion by Sopiruch Yuthawong. The pathological data collection was provided by Sakda Waraaswapati. Mix Wannasarnmetha did manuscript critiquing. Warinthorn Phuttharak did the final manuscript editing. Mix Wannasarnmetha is responsible for the corresponding author.

We would like to express our sincere thanks the statistics advisor, Jitjira Chaiyarit for her statistical assistance.
